# A Brain-Machine-Muscle Interface for Restoring Hindlimb Locomotion after Complete Spinal Transection in Rats

**DOI:** 10.1371/journal.pone.0103764

**Published:** 2014-08-01

**Authors:** Monzurul Alam, Xi Chen, Zicong Zhang, Yan Li, Jufang He

**Affiliations:** 1 Department of Rehabilitation Sciences, The Hong Kong Polytechnic University, Hong Kong, China; 2 Department of Biomedical Sciences, City University of Hong Kong, Hong Kong, China; Instituto de Neurociencias de Alicante UMH-CSIC, Spain

## Abstract

A brain-machine interface (BMI) is a neuroprosthetic device that can restore motor function of individuals with paralysis. Although the feasibility of BMI control of upper-limb neuroprostheses has been demonstrated, a BMI for the restoration of lower-limb motor functions has not yet been developed. The objective of this study was to determine if gait-related information can be captured from neural activity recorded from the primary motor cortex of rats, and if this neural information can be used to stimulate paralysed hindlimb muscles after complete spinal cord transection. Neural activity was recorded from the hindlimb area of the primary motor cortex of six female Sprague Dawley rats during treadmill locomotion before and after mid-thoracic transection. Before spinal transection there was a strong association between neural activity and the step cycle. This association decreased after spinal transection. However, the locomotive state (standing vs. walking) could still be successfully decoded from neural recordings made after spinal transection. A novel BMI device was developed that processed this neural information in real-time and used it to control electrical stimulation of paralysed hindlimb muscles. This system was able to elicit hindlimb muscle contractions that mimicked forelimb stepping. We propose this lower-limb BMI as a future neuroprosthesis for human paraplegics.

## Introduction

Spinal cord injury (SCI) is a devastating neuronal dysfunction that affects approximately 2.5 million people worldwide, with over 130,000 new injuries each year [1]. SCI usually occurs due to a contusion on the spinal cord that results from a traumatic fracture or a dislocated vertebra [2]. Paralysis following SCI can completely disrupt the neural communication from the brain to the body, resulting in disability of movements. For incomplete injuries, some sensory-motor functions can be restored by extensive physical rehabilitation [3],[4]; however, current rehabilitation paradigms are limited or fail to restore any function for motor complete injuries [5]. Though considerable progress is made in stem cell research, available clinical solution for motor complete SCI patients is still lacking [1]. In contrast, brain-machine interface (BMI) provides a connection between the brain and an external device such as a computer cursor [6] or a robotic arm [7], [8]; thereby, enabling persons with paralysis to interact directly with the external world through their natural cognition.

In the last few decades, BMI research has been extended to include upper-limb neuroprosthesis, and many studies of human and non-human primates have shown the feasibility of BMI control of upper-limb neuroprosthesis. However, very few studies have been conducted on the development of BMI control of lower-limb neuroprosthesis [9], [10]. Although some studies have shown that locomotive information can be captured from cortical recordings that are potentially available for BMI controls [11]–[14], experimental evidence to restore lower-limb motor functions of paralysed patients using this BMI technology is sparse. Moreover, although informative, all of these studies, except one [13] have recorded motor commands from the brain of non-injured animals. For the development of BMI to enable movement after SCI, this might be a crucial limitation, as there is less or no sensory feedback to the brain after injury.

Most BMI systems record the activity of hundreds of neural cells and run complex computer algorithms to decode cortical commands [15]–[17]. However, there is still a debate as to whether this approach is better than recording a small number of neurons with their direct control in operating the prosthesis [18]. A recent study, for example, demonstrated that a monkey could voluntarily control a computer cursor by continuously modulating the firing of a single cortical neuron [19]. It remains unknown, however if cortical recording from a small population of neurons can provide information on the gait cycle and whether this information might be used to drive a lower-limb BMI to restore locomotor function after paralysis. As a first step in this direction, in the current study, we address these two major questions in the rodent model of SCI.

We used spinal rats to design and develop a lower-limb BMI for locomotion with the following aims: 1) To determine if neural activity during locomotion in intact rats could be used to determine the locomotive state (standing vs. walking) and identify the phases of a gait cycle, 2) To determine if neural activity during forelimb locomotion in spinalised rats could be used to determine the locomotive state (standing vs. walking) and identify the phases of a gait cycle, and 3) To test if these cortical recordings could be used in the BMI to stimulate hindlimb muscles. Neurons in the hindlimb areas in the primary motor cortex (M1) of spinalised rats were recorded during forelimb walking on a treadmill and used to decode the intent for hindlimb locomotion. We hypothesised that during forelimb walking there is a cortical *intent* for hindlimb locomotion that is evident in neural firing patterns. Our previous results have shown a high rate of correct prediction of step cycles from neural activity recorded during walking [20]. In the present work, we developed a novel BMI device that processed this neural information in real-time and used it to activate paralysed hindlimb muscles electrically to mimic treadmill walking.

## Materials and Methods

All experiments were conducted in compliance with the guide for the care and use of laboratory animals (National Institutes of Health, Publication No. 86–23, revised 1985). The Animal Subjects Ethics Sub-Committee of The Hong Kong Polytechnic University and the animal regulatory body of the Department of Health in Hong Kong approved all experimental procedures. The experimenters held personal licenses for animal surgery. All the experiments were reported in accordance with the ARRIVE (Animal Research: Reporting of *In Vivo* Experiments) guidelines.

### Subjects

Six healthy female Sprague Dawley rats weighing 250–350 g served as subjects in this study. The rats were housed in the Centralized Animal Facilities at the Hong Kong Polytechnic University at a constant temperature of 22°C and on a regular 12 h light/dark cycle. Diet was carefully maintained to keep body weight within a suitable range.

### Treadmill training

Over the first few days of the study, the rats were introduced to a custom-modified treadmill that had three chambers of different widths for walking, separated by acrylic plastic. The rats practiced walking on the moving treadmill belt at different speeds (6.67–10 cm/s) for 1 min. The walking sessions were separated into less than 1 min break for rest. Generally, each rat underwent 10 to 30 walking sessions (each session lasted for 1 min). Each walking session was separated by a rest period of 1 min. Food and water rewards were delivered at the front of the treadmill to keep the rat interested and facing forward. To speed up the learning process, two rats were placed into two different chambers at the same time: one that had already mastered the walking task and one that was new to the task. The treadmill was remotely controlled from outside of the experiment room. A video camera (Pleomax, Samsung C&T Corporation, Korea) was utilised to monitor and record the walking task.

### Microwire array

**Figure 1:**
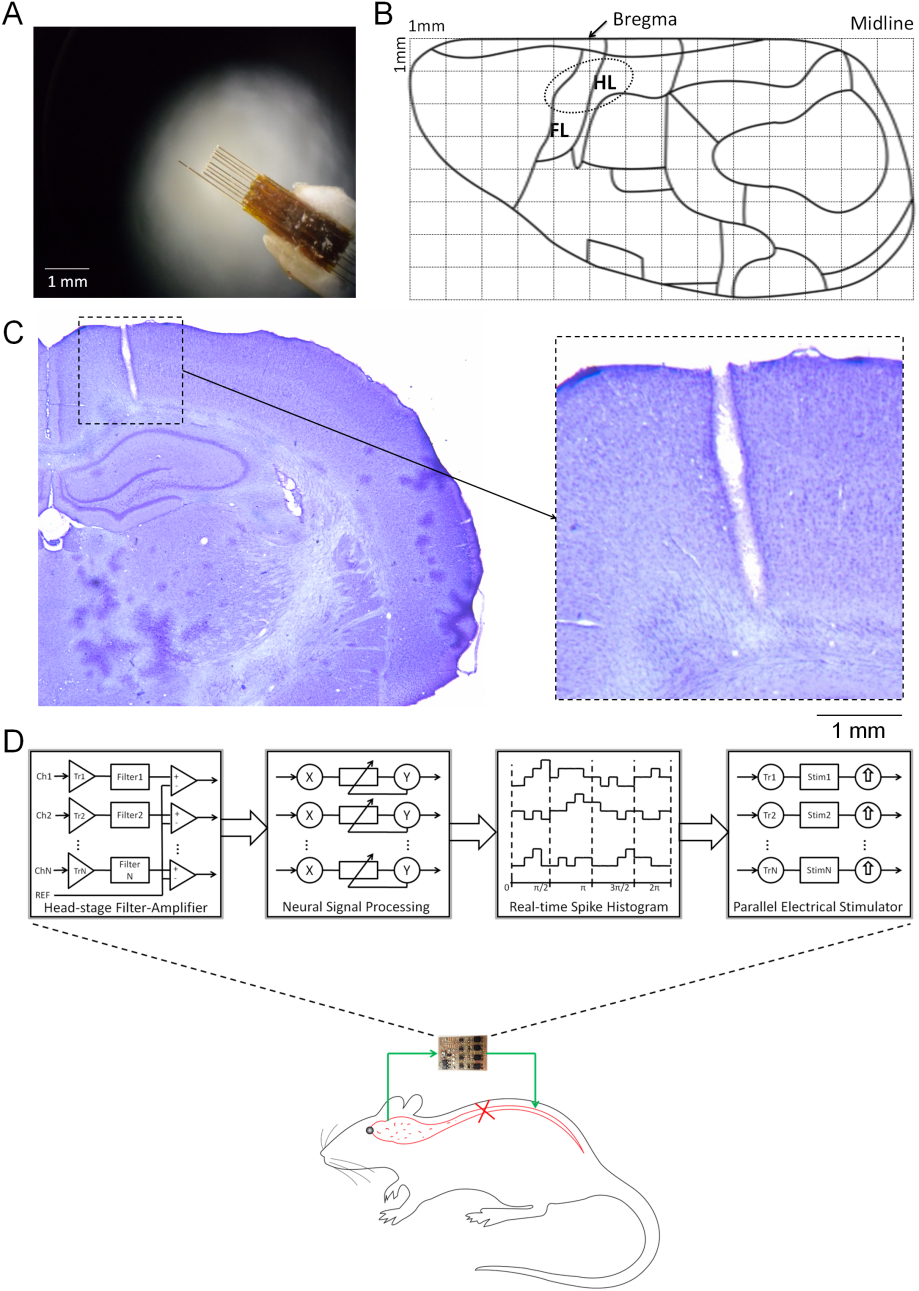
Photograph (A) of the electrode array. (B) Schematic of the hindlimb area of the primary motor cortex (M1). HL: Hindlimb; FL: Forelimb. Dotted area indicates array location. (C) Histological confirmation of the cortical implant of a microwire (*left)* along with a higher magnification view (*right).* (D) Conceptual diagram of the Motolink spinal neuroprosthesis. The cross on the rat schematic indicates a complete spinal transection. The green arrows show the Motolink circuit that allows neural signals to bypass the spinal transection.

We developed custom microwire arrays for recording and stimulation (Figure 1A). Each microwire array was composed of seven recording electrodes, one stimulation electrode, one reference electrode, and one common terminal wire. Recording and reference electrodes were Teflon-coated tungsten microwires (A-M Systems, USA), and the stimulation electrode was stainless steel. Nine polymethyl methacrylate tubes (Polymicro Technologies, USA) were glued-fixed with 500 µm spacing onto a paper base, and a microwire (length, 6 mm) was inserted and glued into each polymethyl methacrylate tube. The reference electrode was kept about 500 µm longer than the recording and stimulating electrodes, and 200–300 µm at the tip of the reference electrode was exposed. Conductive metal wire was utilised as the common terminal of the electrodes. All microwires from the array and the common wire were soldered to a 20-pin female connector (two rows, 1.27 mm pitch) and insulated with silicon glue (Tec-bond 240, Power Adhesives Ltd., UK).

### Cortical implantation

All surgeries were performed under aseptic conditions. Animals were pretreated subcutaneously with atropine sulphate (0.05 mg/kg, Sigma, USA) to restrain tracheal secretions. About 10 min after atropine injection, anaesthesia was induced intraperitoneally with 50 mg/kg pentobarbital sodium (54.7 mg/ml solution, Ceva Sante Animale Co., France) and maintained by supplemental doses (10 mg/kg/h, i.p.). The subject was mounted in a stereotaxic device and body temperature was controlled and maintained between 37 and 38°C by a homeothermic system (Harvard Apparatus, USA). After making a midline incision in the scalp, a small craniotomy (approximately 3 mm×2 mm) was performed across the bregma line. After carefully removing the dura, the primary motor cortex (M1) was mapped by intracortical microstimulation (ICMS) [21], [22] to find the hindlimb motor area. A microwire array was carefully inserted (100 µm/min) into the hindlimb area located +1 to −1 mm rostrocaudal, +1 to +2 mm mediolateral of the left M1 using a remotely operated stepping-motor micro-manipulator (Narishige Group, Japan). The implant location of the array is shown by the dotted area in Figure 1B. Six metal screws were inserted into the skull, and the ground wire was wrapped around one of the screws. Neural recording and stimulation was conducted through the 20-pin socket to which the microwire array was connected. Continuous recording during the insertion of the array was accomplished using an amplifier (A-M system differential AC Amplifier Model 1700, USA) with band-pass filter (0.3–5.0 KHz). The position of the array in the hindlimb area was confirmed by electrical stimulation and visual observation of the movements of right hindlimb. The opening of the skull was covered with a thin layer of silicon epoxy (World Precision Instruments Inc., USA), and a layer of dental cement (Durelon Carboxylate Cement, Germany) was used to cover the microwire array, screws, cables, lower portion of the socket and exposed skull. Histological analysis performed at the end of all experiments show that the recordings were carried out from the hindlimb area of M1 (Figure 1C). The arrow indicates the electrode incision. As the reference electrode was 0.5 mm longer than the recording electrodes, the incision reached the very end of the cortex (tip of reference electrode), while the recordings were mainly from layer V/VI in the motor cortex.

### Neural recording during walking

After 2–3 days of recovering from the surgery, rats were placed onto the treadmill for adaptation. Each rat performed 5–10 practice trials of 1 min length at constant speed (optimum treadmill speed was set according to each subject’s walking speed). Neural signals were recorded using an amplifier (A-M system differential AC Amplifier Model 1700, Sequim, USA) connected to the head connection, and a miniature (1 cm×1 cm×0.4 cm) three-dimensional accelerometer (ADXL335, Analog Devices Inc., USA) was attached to one hindlimb. The accelerometer was used to identify the step cycles during gait. 10–20 recording sessions each of 1 min length were performed. During all recording sessions continuous video records were made for further analysis using a video camera (Pleomax, Samsung C&T Corporation, Korea). Data were digitised at 25 KHz using Axon Digidata 1440 (Molecular Devices Co., USA) and visualised in real time (Axon scope 10.0, Molecular Devices Co., USA) during each experimental session.

### Spinal transection and implantation of stimulation electrodes

After the treadmill experiments in intact rats, a second surgery was conducted to transect the spinal cord. Animals were pretreated subcutaneously with atropine sulphate (0.05 mg/kg, Sigma, USA) to restrain tracheal secretions. About 10 min after atropine injection, anaesthesia was induced intraperitoneally with 50 mg/kg pentobarbital sodium (54.7 mg/ml solution, Ceva Sante Animale Co., France) and maintained by supplemental doses (10 mg/kg/h, i.p.). The animal was mounted in a stereotaxic device and body temperature was controlled and maintained between 37 and 38°C by a homeothermic system (Harvard Apparatus, USA). Laminectomy was made to expose the spinal cord at T9/T10 using a dental drill (Microtorque II, Ram Products Inc., USA). The spinal cord was transected with a microscissor. Care was taken not to damage the spinal arteries. Complete transection was confirmed by the absence of hindlimb movements in response to microstimulation in the cortex.

Custom-made stimulation electrodes were implanted into the hindlimb superficial muscles that are utilized during walking; mainly hip and knee extension and flexion (gluteus superficialis, biceps femoris and semitendinosus). The connecting wires were led subcutaneously to the head socket. The stimulation reference electrode was placed near the lumbar spine. The spinal opening and the skin incisions were sutured carefully.

The animals were administered analgesia of buprenorphine (0.1 mg/kg s.c., twice a day, maintained for 3 days) and antibiotics of penicillin (50,000 units/kg i.m., once a day, in case of infection) after the surgery. The recovery of the subjects was carefully monitored for 3–4 days before starting the treadmill experiments.

### Neural recording during forelimb walking

After 3–4 days of recovery from the second surgery, rats were placed on a treadmill with a harness. The posterior part of the animal was lifted by a custom-made body weight support system, and only forelimb walking was permitted on the moving treadmill belt. Neural signals were recorded using the same amplifier (A-M system differential AC Amplifier Model 1700, Sequim, WA) with band-pass filter (0.3–5 KHz). 10–20 recording sessions each of 1 min were performed. The signals were digitised at 25 KHz using an Axon Digidata 1440 (Molecular Devices Co., Chicago, IL). Continuous video records were made using a video camera (Pleomax, Samsung C&T Corporation, Korea).

### Electronic spinal bridge

We developed an electronic spinal bridge called Motolink to provide a connection between M1 and the hindlimb muscles after spinal injury (Figure 1D). The circuit consisted of three major parts: 1) a head-mountable, multichannel, neural recording amplifier, 2) a real-time neural signal processor, and 3) a controlled electrical stimulation circuit. This circuit filtered and amplified the neural information, then processed this information in real-time to generate trigger signals for the stimulation circuit.

### Multichannel neural signal recording amplifier

**Figure 2:**
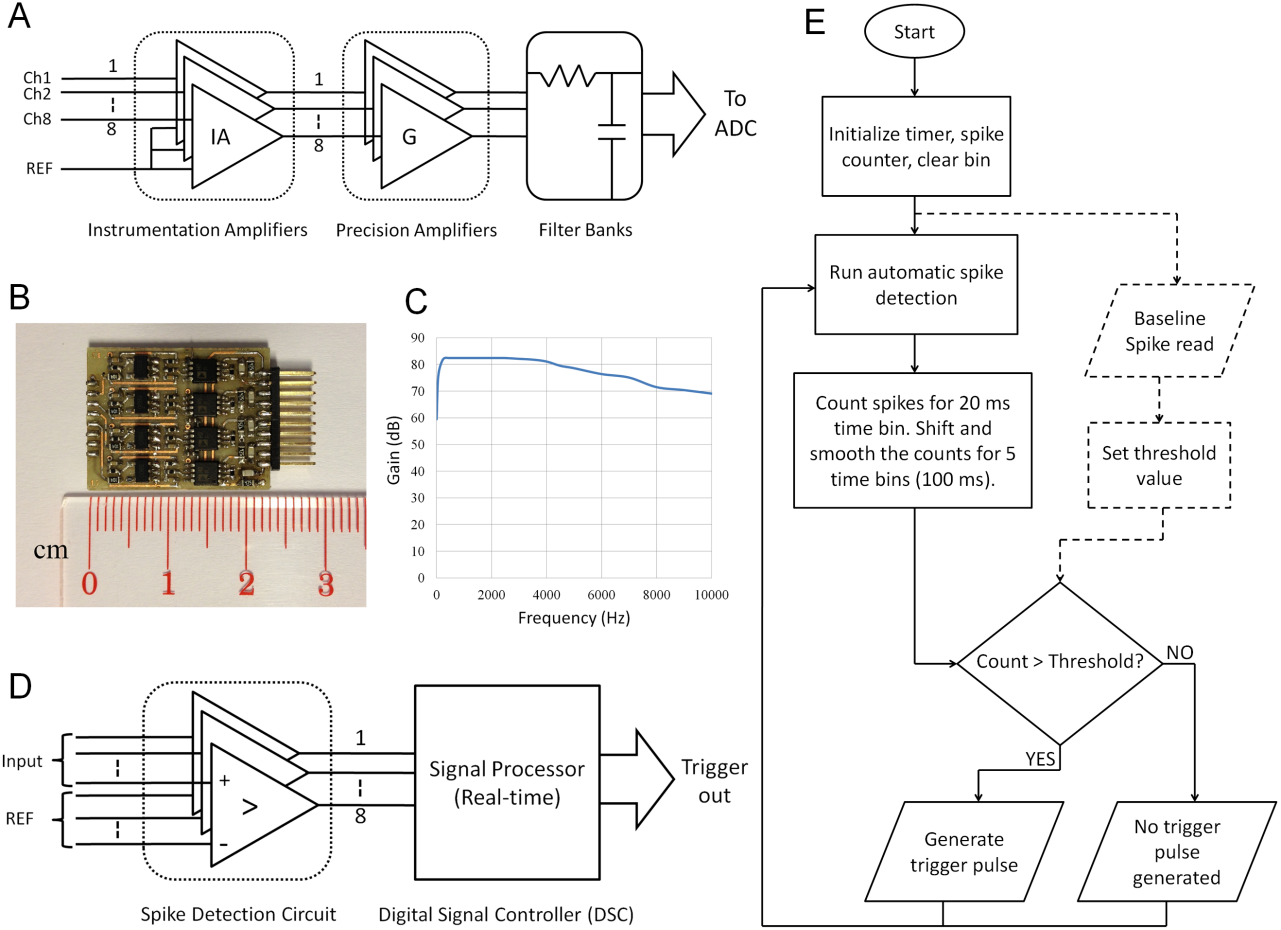
Hardware details of the Motolink system. (A) A block diagram of the amplifier circuit. (B) A photograph of the complete circuit board. (C) The frequency response of the recording amplifier. (D) A block diagram of the neural signal detection and processing circuit. (E) A diagram of the program flow chart.

The analogue front-end circuit is one of the most critical parts of any electrophysiology system [23]. Due to space limitations, we considered commercial off-the-shelf (COTS) integrated circuits with small foot-prints. The front-end consisted of two main stages: the preamplifier stage, built with an instrumentation amplifier (IA), and a second-order Sallen-key filter amplifier, constructed with a low-noise operation amplifier. Figure 2A shows the block diagram of the amplifier circuit.

For successful recording of neural spikes, it is important to keep the noise level as low as possible [24]. Noise is an inherent characteristic of electrical circuits and is associated with the random nature of the movements of charge carriers [25]. When measuring very small signals with an IA, it is important to consider the different sources of noise to choose the correct amplifier. Noise is a Gaussian distribution of voltages centred at zero, and is represented in terms of root mean square (RMS) power [26], where all noise sources are added together in a root sum of squares fashion as follows:
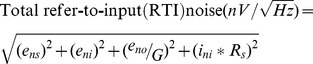
Here, e*_ns_* refers to source resistance noise, e*_no_* and e*_ni_* refer to the voltage noises, and i*_ni_* refers to the current noise of the IA.

We chose the AD8220 (Analog Devices Inc., USA) IA for our design due to size advantages. The typical electrode impedance is generally 1 MΩ for electrophysiological recordings [27]. Using this optimum value and with additional data from the IA datasheet, we calculated the noise for our designed amplifier to be 129.3 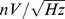 based on the following formula:
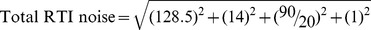



This is nearly identical to the noise with only the source resistance. The total RMS noise can be limited by filtering after the IA to reduce the bandwidth as much as possible without distorting the signal of interest. Noise at these later stages is less significant because the RTI noise value is divided by the gain of the IA. In our design we obtained an effective noise bandwidth from 300 Hz to 5 KHz. For our two-pole low-pass filter with a cut-off frequency (f_C_) of 5 KHz, this resulted in an effective noise bandwidth of 1.11*f_C_. We used this approximation to calculate overall RMS voltage noise of the system as follows:
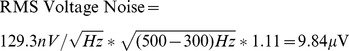



When recorded by microwire arrays, the amplitude of extracellular spikes is generally 100–300 µV [27]. Hence, 10 µV instrument noise is sufficient for the recording of neural spike activities. The complete printed circuit board of the analogue front-end circuit (Figure 2B) was small enough to be mounted on the head of a rat. Figure 2C shows the frequency response of the amplifier. The pass-band gain of the circuit was just over 80 dB. An advantage of this design is the ease with which the cut-off frequencies can be manipulated for other experimental requirements.

### Neural signal processor

We developed a neural signal processing system to decode the locomotive information contained within the neural signals in real-time. The system was composed of two major parts: an online spike detection circuit and a signal processor unit (Figure 2D).

We installed and tested different online spike detection algorithms. Among them, the median value spike threshold method was the best that matches the finding of a previous study [28]. The threshold for each channel was determined in a spike training phase according to the following equation:
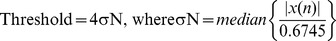



The processing of neural signals was accomplished by a 16-bit digital signal controller that was fast enough to process several neural channels simultaneously. Large on-chip memory permitted spike counts to be made in 100 ms bins. The bin was moved every 20 ms to generate a smooth spike histogram, as shown by the program flowchart in Figure 2E. After initialization (setting up a counter and time bins) the neuroprocessor runs baseline firing rate calculation of a given signal to calculate the threshold value (number of spikes per 100 ms) and stored in the Motolink system’s permanent memory. This task only executed at the very first time when the system was powered up, as shown in Fig. 2E (dotted). Every time afterward, the system went to the general loop (Fig. 2E, solid). In this loop, the processor ran automatic spike detection and counting of real-time signal. To produce reliable output, we had selected the best recording channels from the electrode array. For each recording channel the processor counted the number of spikes for every 20 ms time bin. Total 5 time bin counts were stored in the working memory of the Motolink system. The counted values in these time bins were smoothed by a moving average before comparing with the threshold value. When the smoothed average count was higher than the pre-stored threshold count, the neuroprocessor generated a trigger signal for the stimulation circuit. This online counting, comparing and triggering loop continued as long as the system was powered ON.

### Controlled electrical stimulation circuit

We used biphasic pulses to stimulate the muscles. Firstly, biphasic voltage pulses were generated by a stimulator (Master-8 A. M. P. I., Israel) from the external trigger of our neural signal processing system. To isolate our recording system from the electrical stimulation, we then utilized a current isolator for each stimulation channel (Harvard Apparatus, USA). The current isolator works as a voltage-to-current converter circuit that generates 100 µA of biphasic currents per volt of stimulation. To find the optimum stimulation parameters, we varied the amplitude (1–10 V), width (200–500 µs), frequency (10–100 Hz) and stimulation pulse number (1–5) in anesthetized subjects. During the experiment sessions, except the amplitude, these parameters were kept constant. The amplitude generally had to be increased as the experiment progressed in order to generate hindlimb movements from this stimulation. We found that 100 Hz stimulation with 0.5 ms stimuli of 3 pulses elicited rhythmic bilateral hind leg movements as demonstrated in Video S1.

### Histology

After all experiments were completed, the animals were deeply anaesthetised intraperitoneally with 60 mg/kg pentobarbital sodium (54.7 mg/ml solution, Ceva Sante Animale Co., France). A constant direct current (approximately 100 µA) was delivered for about 1 min to each of the electrodes to produce heat markers at the implanted areas. The animals were then perfused transcardially with 300 ml 0.9% sodium chloride, followed by 300 ml of ice-cold 4% paraformaldehyde in 0.1 M phosphate buffer (pH 7.4). The brain was removed and post-fixed for 24 h in the same fixative. The brain was then dehydrated in 30% sucrose for three days. Coronal sections (50 µm) of the brain were sliced in cryostat microtome and Nissl staining was performed on M1 cortical slices. Observation of these stained slices was carried out under a microscope (Nikon Corporation, Japan) and pictures were captured and stored on a computer.

### Data analysis

The data were analysed offline using custom-scripts written in MATLAB (MathWorks, Nitick, USA). Extracellular data were processed utilising Axon Clampfit 10.0 (Molecular Devices Co., Chicago, USA). Neural spikes were detected and sorted using a MATLAB-based open source electrophysiological data processing toolbox [29]. Neural spikes rate were compared with the reference time signals from the accelerometer to determine the relation between step gait cycles and neural spikes. Neural activity was studied during the transition from stand-to-walk and from walk-to-stand. Neurons active in the stand-to-walk transition were named *Start* units, and neurons active in the walk-to-stand transition were named *Stop* units. For each unit, spikes were first binned into 50 ms and then a perievent time histogram (PETH) was built by using the transition point from stand-to-walk as time 0. The mean spike rate from 1.5 s to 1 s before the transition time (stand-to-walk) was used as the baseline. For each 50 ms bin in this PETH, normalized firing rate values were calculated by subtracting the mean firing rate of the baseline and then by dividing it by the standard deviation of the baseline. Finally, normalised firing rates of all *Start* and *Stop* units were generated, and units were grouped according to their firing rate patterns. For step gait cycles each step time was first converted into percentage. A full cycle represents from 0 to 100%; where 30% is stance phase and the rest 70% is swing phase [30]. Then spikes were binned into each 5% (18°) and then a phase histogram (PH) was built for each unit. Raster plots and spikes time histograms were also generated for each detected unit. Neural spikes rate were compared before and after spinal transection to determine the changes in cortical activity that may have occurred due to spinal transection. Firing rats before and after SCI during standing and walking were analysed using a pair t-test.

## Results

### Neural activity during walking in intact rats

**Figure 3:**
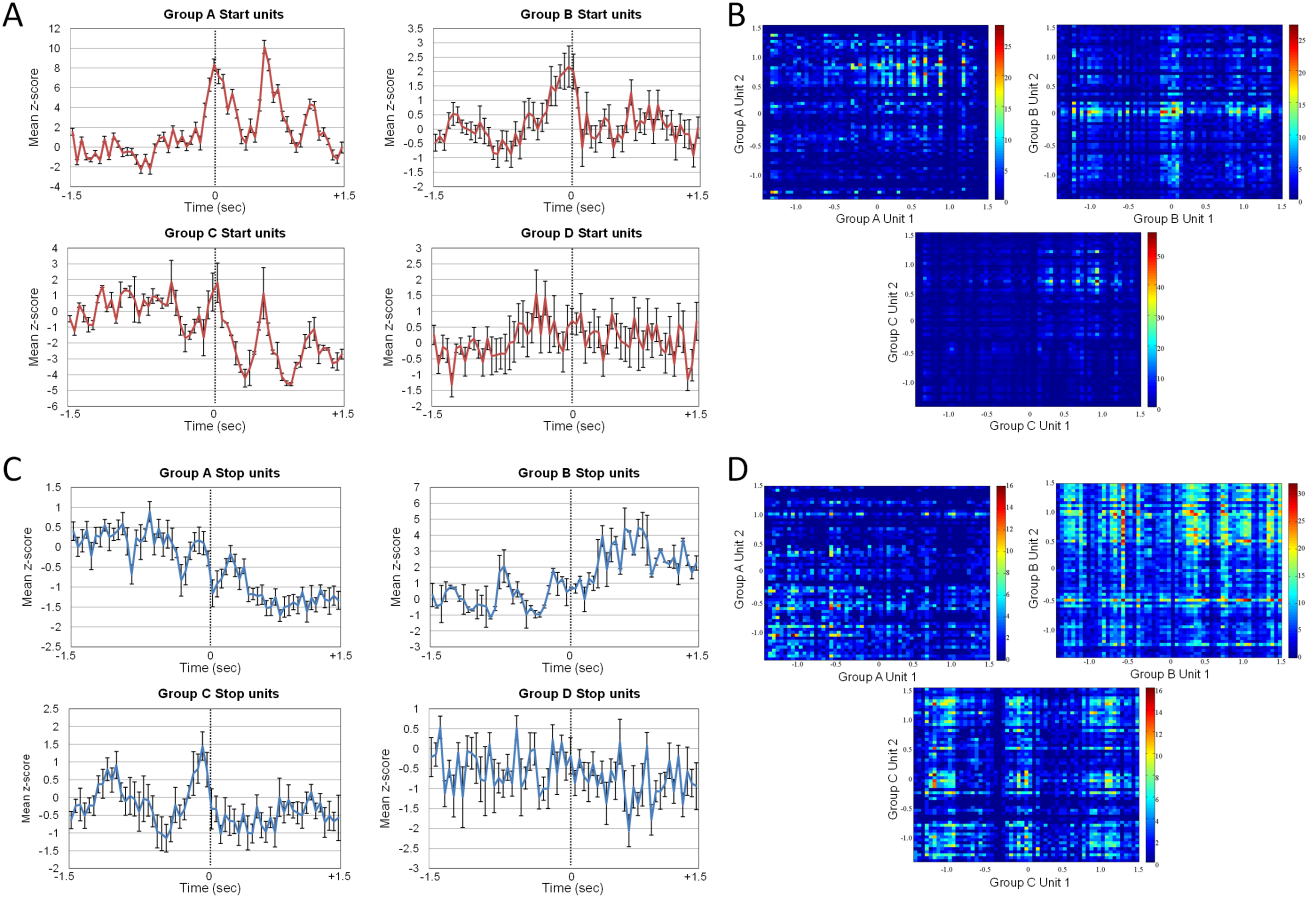
Neural spikes during stand to walk and walk to stand transitions. (A) The mean z-scores for each of the four firing rate patterns observed in the transition from standing to walking in intact rats. 0 is the transition time from standing to walking. (B) Correlation between units within each group (Group A, B and C Start units). (C) The mean z-scores for each of the four firing rate patterns observed in the transition from walking to standing in intact rats. 0 is the transition time from standing to walking. (D) Correlation between units within each group (Group A, B and C Stop units).

We detected 10–20 isolated single units from each animal. A total of 80 units were analysed in the present study. Four groups of *Start* units were identified (Figure 3A). Units in group A (20 units, 25% of the single units identified) had three positive peaks in firing rate at the onset of walking, units in group B (26 units, 32.5% of the single units identified) had a single positive peak in firing rate at the onset of walking, units in group C (8 units, 10% of the single units identified) had three positive peaks in firing rate at the onset of walking and a decrease in baseline firing rate; and units in group D (26 units, 32.5% of the single units identified) had no significant change in firing rate at the onset of walking. Correlations between the firing rates of *Start* units in different groups (A, B and C) are shown in Figure 3B.

Similarly, four groups of *Stop* units were identified (Figure 3C). Units in group A (30 units, 37.5% of the single units identified) had a decrease in baseline firing rate at the onset of standing, units in group B (8 units, 10% of single units identified) had an increase in baseline firing rate at the onset of standing, units in group C (16 units, 20% of single units identified) had a positive peak in firing rate at the onset of standing; and units in group D (26 units, 32.5% of the single units identified) had no significant change in firing rate at the onset of standing. Correlations between the firing rates of *Stop* units in different groups (A, B and C) are shown in Figure 3D.

**Figure 4:**
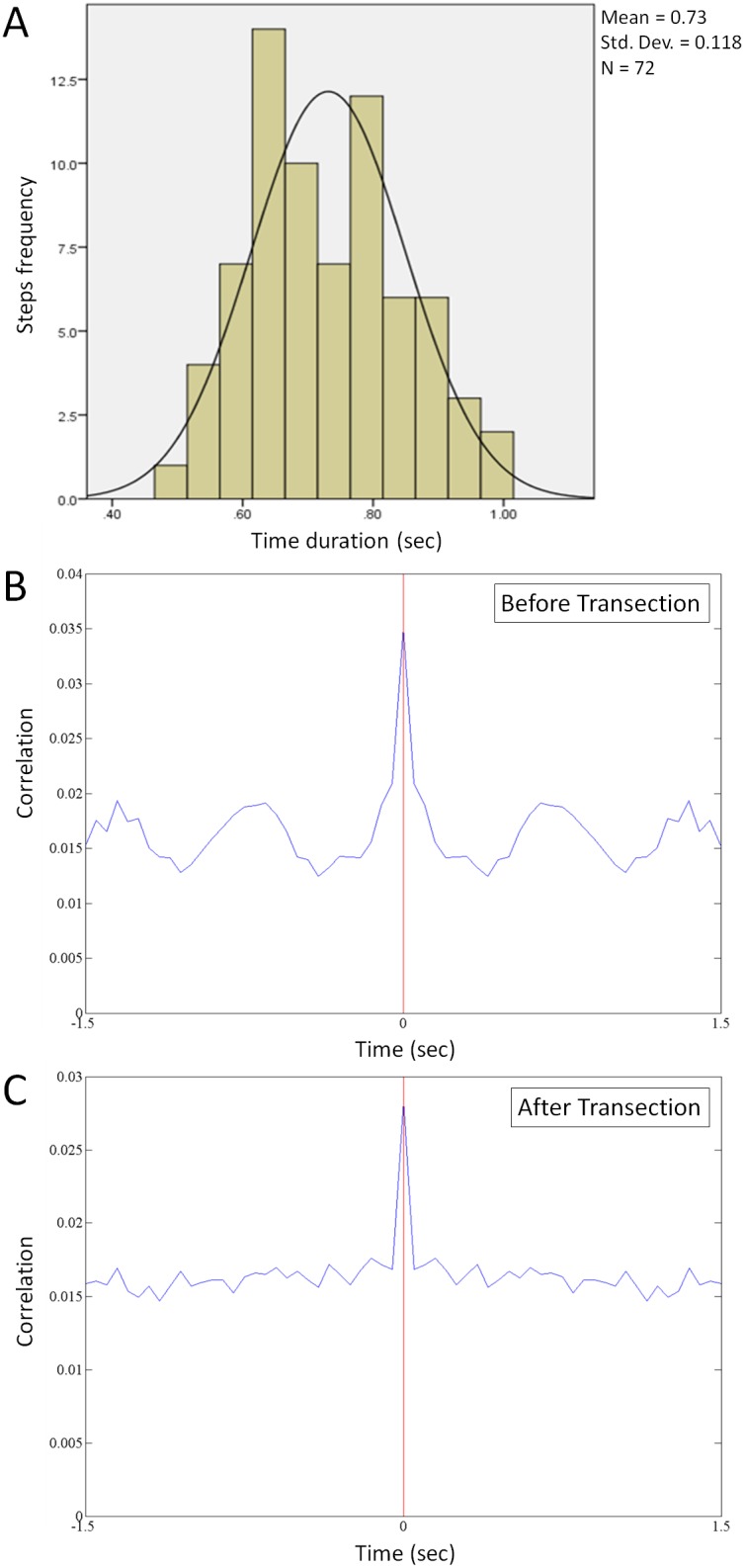
Step time histogram (A) with a fitted distribution curve of walking indicates average one step time of 0.73 s (SD: 0.118 s) in healthy subjects. (B–C) The correlation of a neural spike recorded during walking before (B) and after (C) spinal cord transection.

**Figure 5:**
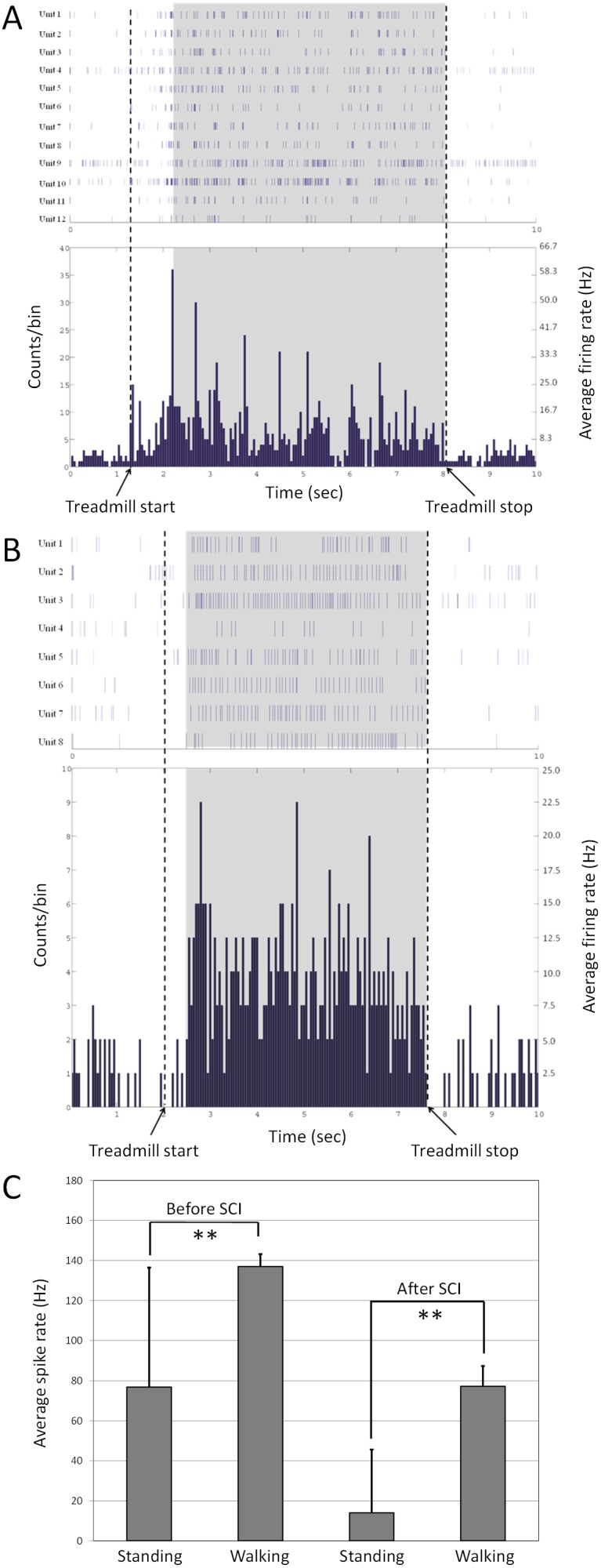
Neural spikes during standing and walking in healthy and spinal subjects. (A) Spike rate of 12 cortical neurons from a single rat recording during walking on a treadmill before spinal cord transection (SCT). The top panel illustrates the time raster of each unit, and the bottom panel illustrates the total spike counts per 50 ms time bin. (B) Spike rate of eight cortical neurons recorded from a rat during forelimb walking on a treadmill after SCT. The top panel illustrates the time raster of each unit, and the bottom panel illustrates the total spike counts per 50 ms time bin. (C) The average spike rates of all neurons recorded during standing and walking from all rats before and after SCT (**p<0.01, paired t-test).

**Figure 6:**
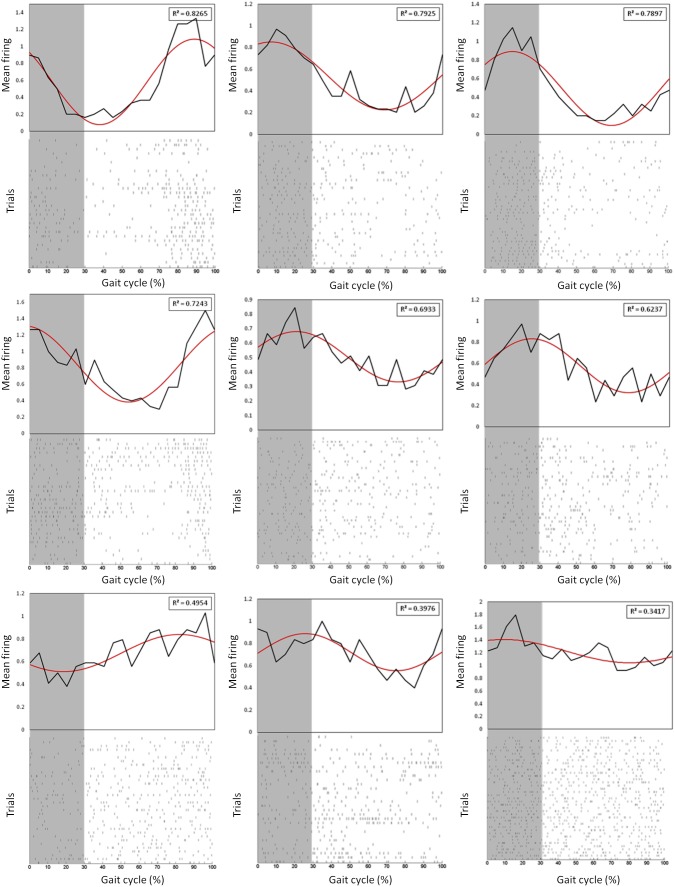
Neural spikes during different phases of walking. Each panel represents one neural unit. The upper panel shows the mean firing (black line) expressed relative to the step gait cycle in intact rats, and the lower panel shows the raster plot. A sinusoidal fitting line is shown in red and the adjusted R-square value for this fitting is presented in each panel. The grey area is swing phase and the rest is the stance phase of one gait cycle expressed as percentage.

The mean one step walking time was 0.73 s (standard deviation 0.118 s; Figure 4A). Total 72 steps were analysed during the treadmill walking task. Over 65% of the steps appeared in between 0.62 to 0.85 s; while all the steps were ranged in between 0.49 to 1 s. A normal distribution curve fits almost all the step times. In each animal, 10–20 single units were identified during walking. Figure 5A shows the average firing rate of these units for 10 s. The walking started 2.5 s after standing, and stopped 5 s after the transition from walking to standing. The firing rate was generally higher during walking. Some neurons (2–3 in each subject) demonstrated rhythmic modulation of firing rate. In each step, these neurons tended to fire in specific gait phase angles (Figure 6). These neurons are considered as having the best rhythmic patterns (top panels with adjusted R-square value >0.50) during walking, as the rhythmic modulation of firing rate was fitted with a single sinusoidal wave (Figure 6, red lines). The neurons with lower coefficients (adjusted R-square value <0.50) of firing rate modulation during walking were also analysed. These neurons also tended to fire in specific gate phase cycles but with lower significance (Figure 6; lower panels).

### Neural activity during forelimb walking in spinalised rats

After spinal cord transection animals performed a forelimb walking task with suspended hindquarters on the same treadmill. The previously identified active units were dropped in all animals. In each animal, 7–10 single units were identified and sorted from the cortical recordings. Figure 5B shows the average firing rate of these units for 10 s. Similar to before the spinal cord transection, the walking started 2.5 s after standing, and stopped 5 s after the transition from walking to standing. The highest firing rate (∼22 Hz) occurred on two occasions, both during walking. The average firing rate during standing and during walking was lower after spinal cord transection (14 Hz during forelimb standing and 77 Hz during forelimb walking) than before spinal cord transection (77 Hz during standing and 137 Hz during walking; both p<0.01, paired t-test; Figure 5C). However, it was still possible to detect locomotive states (standing or walking) from these recordings. No neurons identified after spinal cord transection demonstrated rhythmic modulation of firing rate (Figure 4B–C).

**Figure 7:**
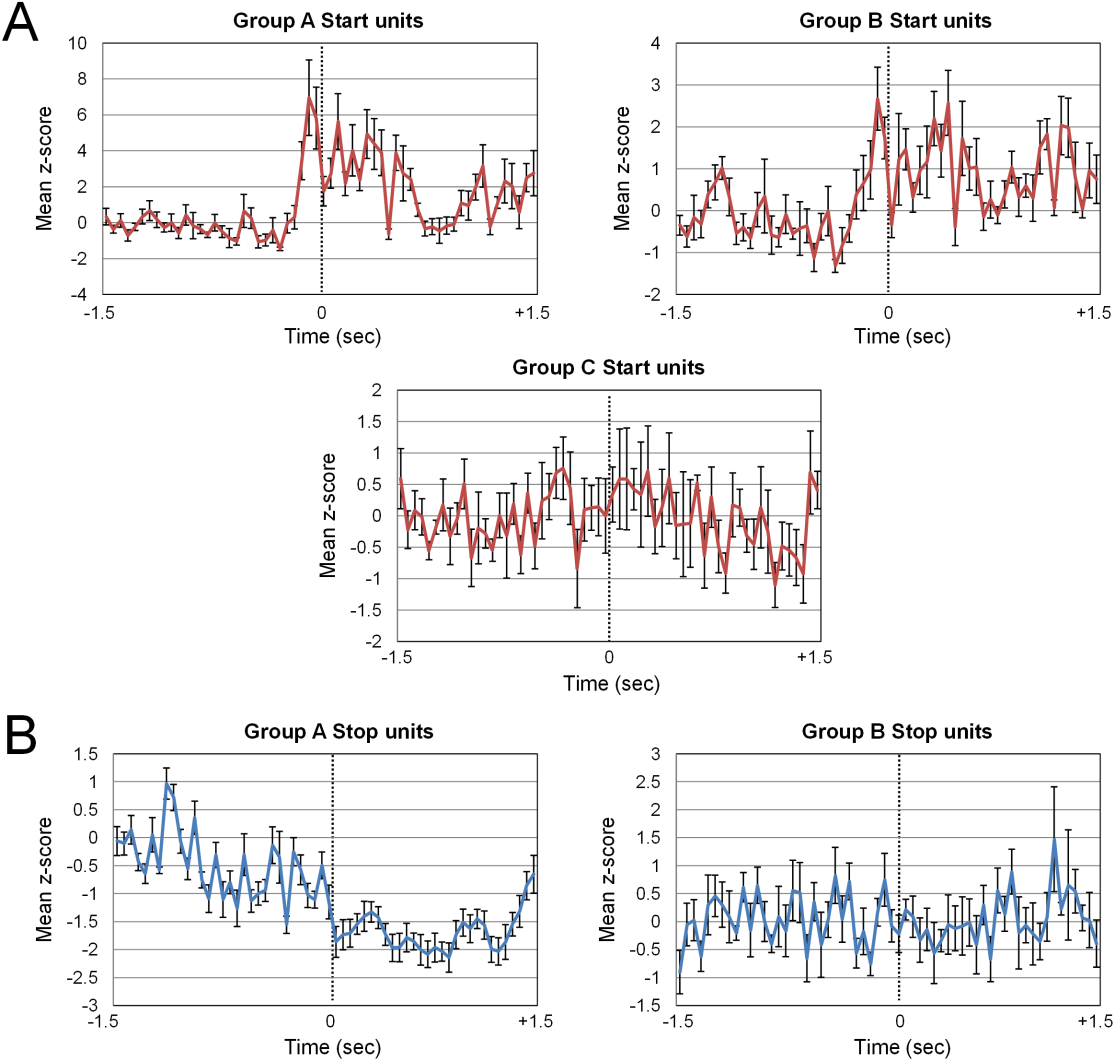
Neural spikes in standing and walking transition after spinal transection. (A) The mean z-scores for each of the three firing rate patterns observed in the transition from standing to walking in rats after spinal cord transection. 0 is the transition time from standing to walking. (B) The mean z-scores for each of the two firing rate patterns observed in the transition from walking to standing in rats after spinal cord transection.

Three groups of *Start* units were identified in the neurons active during forelimb walking after spinal transection (Figure 7A). Units in group A (24 units, 30% of the single units identified) had a positive peak in firing rate at the onset of forelimb walking, units in group B (24 units, 30% of the single units identified) had a slight increase in baseline firing rate from the onset of forelimb walking, and units in group C (32 units, 40% of the single units identified) had no significant change in firing rate at the onset of forelimb walking. Two groups of *Stop* units were identified (Figure 7B). Units in Group A (56 units, 70% of the single units identified) category had a decrease in firing rate from the onset of standing, and units in group B (24 units, 30% of the single units identified) had no significant change in firing rate at the onset of standing. From these recordings, it was still possible to detect the start and stop times of walking.

### Triggering electrical stimulation from direct cortical recordings

**Figure 8:**
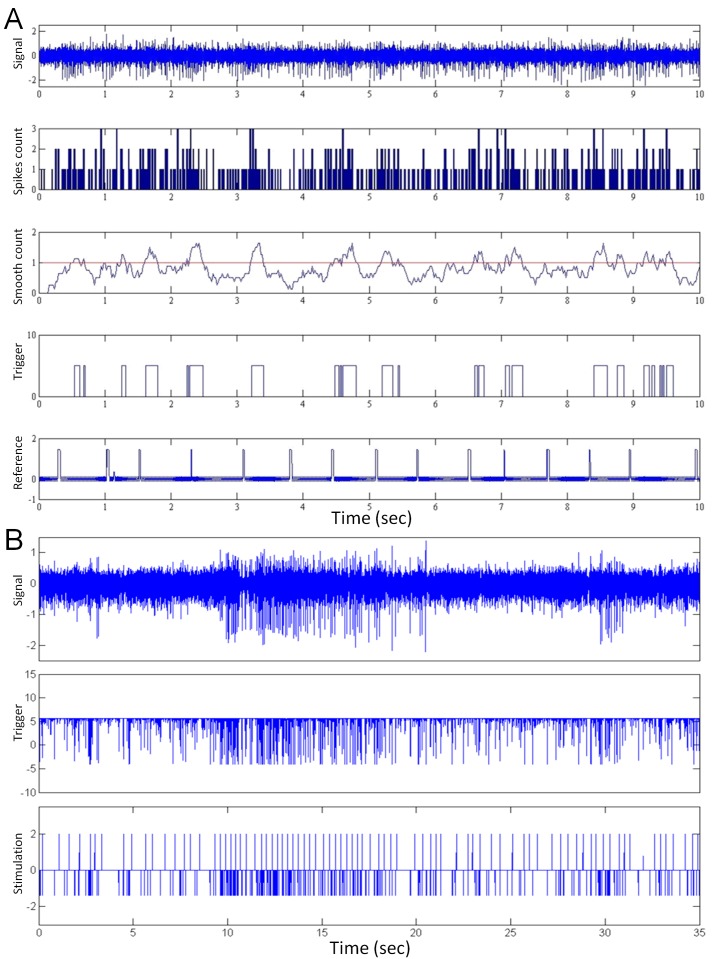
Electrical stimulation algorithm test, (A). Y-axis in each panel represents voltage. A raw neural signal recorded from one cortical electrode is shown in the first panel. The signal was processed to generate spike histogram and then smoothened. The smoothened signal was then compared with the threshold (shown by red line) to generate the voltage triggers. The final panel shows the original reference signal from the accelerometer during treadmill walking. (B) Frequency modulation of cortically triggered stimulation in a freely roaming rat. Y-axis in each panel represents voltage. The Motolink hardware generates trigger from the raw signal. The final panel shows the biphasic stimulation signal generated from the trigger signal.

To test the performance of Motolink, offline emulation was performed for a single channel cortical recording (Figure 8A). The neural detection and processing circuit (Figure 2D) processed a raw neural signal that had been recorded from one cortical electrode during gait of a healthy rats, and generated a spike histogram (Figure 8A, panel two). After smoothing and thresholding (Figure 8A, panel three), emulated triggers were generated (Figure 8A; panel four). The predicted step times were calculated, and compared to the actual step times observed during gait (Figure 8A, panel five). 11 out of 14 steps were successfully identified (79% accuracy) with a mean time delay of predicted steps from the actual steps was 113.41 ms (standard deviation 92.13 ms). There were three miss-triggers, whereby a step occurred but was not predicted, and few false-triggers, whereby a step was predicted but did not occur. We implemented 50 ms delay after each successful step detection in our decoding algorithm. This is a safety routine to prevent unwanted stimulation trigger signals immediately after the preceding signal. This helps protect the hindlegs from overstimulation by possible false triggers.

To test the performance of Motolink in real-time, the hardware was connected to the head socket of the spinalised rat in a cage. After setting the threshold values, the Motolink hardware successfully generated electrical stimulations in real-time. Figure 8B shows a single channel cortical recording, online spike detection and generation of biphasic electrical stimulation pulses from an unrestrained rat. The detected spikes had higher frequency modulation during forelimb crawling and also reflected in the electrical stimulation (Figure 8B, last panel).

Finally, the Motolink hardware was tested in the spinalised rats (n = 6) while they were performing the forelimb walking task on a moving treadmill belt with suspended hindquarters. The cortical signals generated during forelimb walking were used to control electrical stimulation of the hindlimb muscles. Stimulation-induced contraction of the hindlimb muscles occurred during the forelimb movements (Video S1). Figure S1 illustrates the raw neural signals and trigger signals generated by the Motolink hardware. Two trigger signals were generated in real-time from two cortical recording channels for stimulating both hindlimbs. Thus, for the first time, a brain-machine-muscle interface was established for the restoration of hindlimb locomotion in spinal rats.

## Discussion

Our results on intact rats demonstrated that neuronal activity differed according to the locomotive state (standing vs. walking). The transition from one locomotive state to another locomotive state (standing to walking and walking to standing) could be predicted from the patterns of mean firing rates of cortical neurons. Neurons in the same group showed similarities in their responses at different time points during the standing to walking and walking to standing transitions. There was some overlap between *Start* and *Stop* neurons. Some *Start* neurons also functioned as *Stop* neurons. In addition, different gait phase angles could also be predicted from these neural recordings during walking. Thus, during walking performed by intact rats, ample locomotive information could be extracted from cortical recordings. This supports previous findings of task-related modulation of cortical activity during motor tasks in cats [31]–[33] and indicates that different locomotive information (locomotive state, transition and gait cycles) can be simultaneously captured from recordings made from the rat hindlimb M1.

Furthermore, our results indicated that different locomotive states (standing vs. walking) could also be predicted from cortical recordings made after complete spinal transection. However, detailed locomotive information on the timing of gait cycles could not be obtained from cortical recordings made after spinal transection. A recent study may explain this finding, as it showed that in cats, different sources of locomotion (controllers) are located in the spinal cord, rather than in the cortex [34]. After spinal transection, the efferent copy from these limb controllers (spinal network) could not reach the brain [35]. Nonetheless, the identification of different locomotive states (standing vs. walking) after spinal cord transection could still be utilised for the control of neuroprostheses. Indeed, our Motolink hardware was able to utilise this residual neural information to stimulate hindlimb muscles to mimic forelimb walking. Thus for the first time, a brain-machine-muscle interface was established for the restoration of hindlimb locomotion function in spinal rats.

To exclude the possibility of recording the neural units that may be linked to the intact forelimb related motor tasks of spinalised rats, we confirmed the location of electrodes by intracortical microstimulation (ICMS). During electrode implantation, we observed hindlimb movements occurred by ICMS, while there was no forelimb movement. By this way we confirmed that the recordings were only from the hindlimb area of the primary motor cortex. The basic assumption of this study was that the hindlimb cortical area of rat responds to hindlimb related motor functions; however evidence from literature supports this premise [14], [36]. After spinal transection these natural functions disrupted, but left some residual neural activities that could still be used for neuroprosthetic controls. Our Motolink hardware utilizes these residual neural signals to stimulate the hindlimb muscles during forelimb walking (Video S1). However, this does not preclude the possible involvements of other cortical areas in contribution to hindlimb movements [37], [38].

Recent studies on spinal rats demonstrated full weight bearing hindlimb standing and walking with the aid of pharmacology and epidural spinal cord stimulation [39]–[41]. In these studies, the stimulators were externally controlled for the initiation of the locomotor activities, similar to a previous study [42]. Our current study shows that after spinal transection, the residual neural signals can still be used to predict the transition of locomotive states (standing vs. walking). Hence, these cortical signals can be utilized to operate the spinal stimulator, and thus can provide an intention driven locomotion. The distinguished neural spike patterns of stand to walk transition can be utilized to turn-on the spinal stimulator; while the walk to stand transition patterns can be utilized to turn-off the spinal stimulator. Therefore, this method could provide a natural intent driven locomotion after motor paralysis.

In the current study, the Motolink system utilized only one electrode array (implanted unilaterally in one hemisphere of the primary motor cortex), the signal from which was used to trigger the start and stop of both hindlimbs movements. Our current system is useful in identifying walking vs. standing states, but lacks the capability for detecting alternating synchronization of limb movements. An ideal neuroproshtetic system for locomotion will necessitate extracting neural signals bilaterally from both motor cortices. In our future work, we will adapt a Motolink system that utilizes multiple electrode arrays implanted bilaterally to record and decode the neural signals for locomotion via an advanced computational algorithm.

More work is needed to establish the concept of cortically triggered electrical stimulation for the restoration of lower-limb motor functions after paralysis. A better understanding of the cortico-cortical connections in the motor cortex between locations of forelimb and hindlimb; and their neural spikes synchrony during locomotion would assist in developing a more reliable and practical neuroprosthetic system. This is largely because locomotion task differs from other tasks such as forelimb reaching and grasping. Furthermore, one should reinvestigate the neural activities and their possible synchrony for translating these findings to bipeds.

Additionally, there are some very important bioengineering issues that need to be resolved [43]. One of the most important issues is the biocompatibility of electrodes and implants [44], [45]. Compensating for foreign body reactions, such as gliosis has been a big challenge towards the development of long term neural interface system. Researchers are working on this issue to develop advanced biomaterials that satisfy this demand. Neural decoding is another engineering challenge that needs to be addressed [27]. At the moment, the computation cost is too high, which further increases with an increase in the number of recording channels. Although the modern signal processors are mostly capable of achieving this demand, there are drawbacks such as high heat dissipation, large chip size and high power requirement that limit the development of advanced neuroprosthetic devices.

## Supporting Information

Figure S1 **The data in this figure refers to supplementary Video S1.** Y-axis in each panel represents voltage. The stimulator was turned on at 10^th^ s and turned off at 34^th^ s, and the treadmill was running from 15.5^th^ s to 26^th^ s. Two hindlimb triggers (Tr1 and Tr2) were generated from two cortical recording channels (Ch1 and Ch2).
(TIF)

Video S1 **Cortical signals generated during forelimb walking triggered electrical stimulation for hindlimb movements.** The LED light in the video glowed while the stimulation was ON.
(MPG)
