# Supplementary figures and images for: A Brain-Machine-Muscle Interface for Restoring Hindlimb Locomotion after Complete Spinal Transection in Rats
Source: PLoS One. 2014 Aug 1;9(8):e103764. doi: 10.1371/journal.pone.0103764 (PMC4118921; doi:10.1371/journal.pone.0103764)

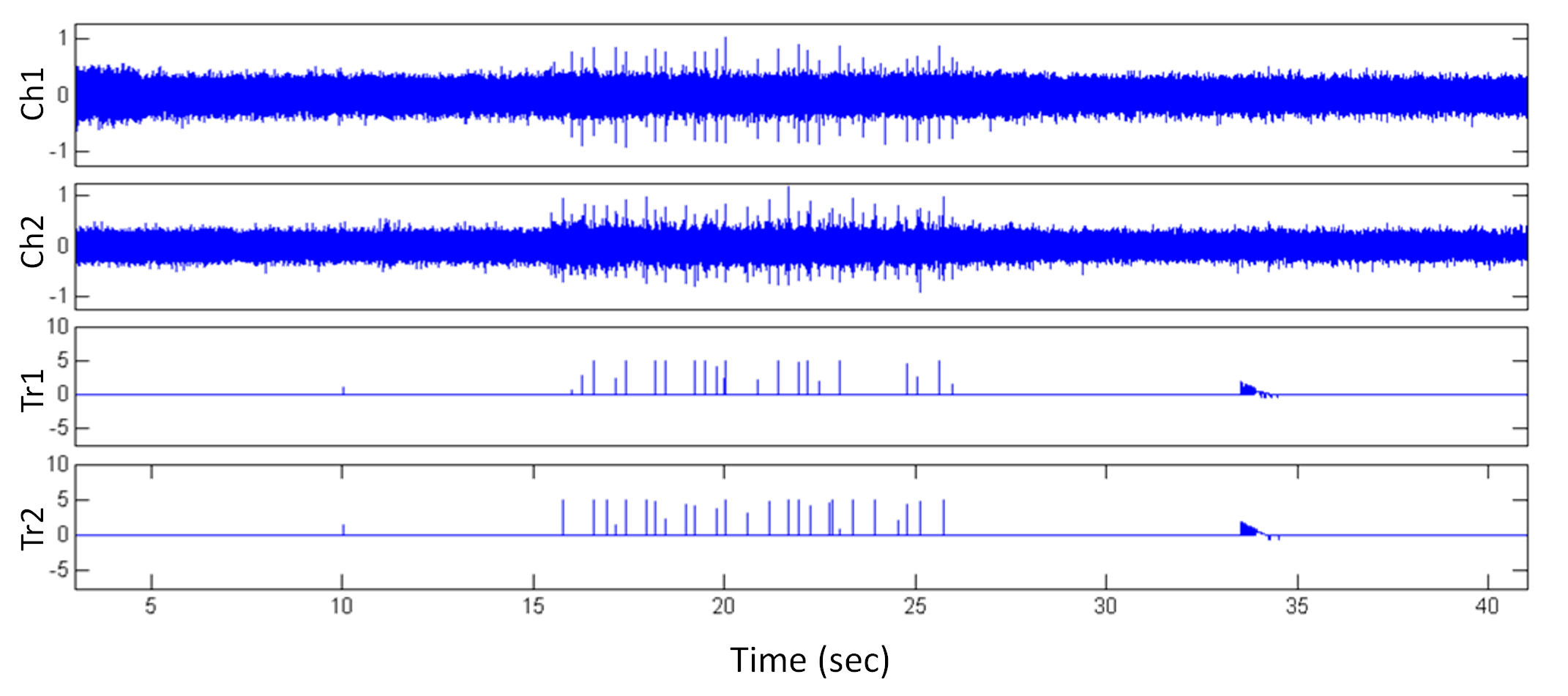

Supplement: Figure S1 — The data in this figure refers to supplementary Video S1. Y-axis in each panel represents voltage. The stimulator was turned on at 10th s and turned off at 34th s, and the treadmill was running from 15.5th s to 26th s. Two hindlimb triggers (Tr1 and Tr2) were generated from two cortical recording channels (Ch1 and Ch2). (TIF) [file pone.0103764.s001.tif]
